# Placental Pathology in preeclampsia and gestational hypertension; A comparative study

**DOI:** 10.12669/pjms.42.9.16844

**Published:** 2026-09

**Authors:** Ruqaiya Shahid, Muhammad Furqan Bari, Jahanara Hasan, Waqas Ahmed Farooqui

**Affiliations:** 1 Dr. Ruqaiya Shahid, FCPS. Associate Professor, Dow International Medical College, Dow University of Health Sciences, Karachi, Pakistan; 2 Muhammad Furqan Bari, PhD. Department of Pathology, Dow International Medical College, Dow University of Health Sciences, Karachi, Pakistan; 3 Jahanara Hasan, PhD. Department of Obstetrics and Gynecology, Dow International Medical College, Dow University of Health Sciences, Karachi, Pakistan; 4 Waqas Ahmed Farooqui, PhD. School of Public Health, Dow International Medical College, Dow University of Health Sciences, Karachi, Pakistan

**Keywords:** Eclampsia, Hypertension, Low Birth Weight, Pregnancy, Placenta, Pre-Eclampsia, Stillbirth

## Abstract

**Objectives:**

To evaluate placental histopathological lesions in hypertensive disorders of pregnancy and their association with maternal and fetal outcomes.

**Methodology:**

This cross-sectional study was conducted in the Departments of Pathology and Gynecology & Obstetrics, Dow Hospital, Dow University of Health Sciences, from January-December 2021. Seventy-six pregnant women were included: preeclampsia (n=29), gestational hypertension (n=24), and normotensive controls (n=23). Placentae were examined microscopically, and lesions were classified according to the Amsterdam criteria into maternal vascular malperfusion (MVM), fetal vascular malperfusion (FVM), and inflammatory lesions. Association with clinical group was assessed by Poisson regression analysis, expressed as incidence rate ratio (IRR).

**Results:**

Placental lesion categories were significantly different across the clinical groups (p=0.018). MVM lesions, including accelerated villous maturation, distal villous hypoplasia, villous infarction, decidual arteriopathy, and low fetal-to-placental weight ratio, were increased in preeclampsia (IRR=4.4, p<0.001) and gestational hypertension (IRR=2.5, p=0.005). FVM showed no association. Inflammatory lesions demonstrated a borderline association with preeclampsia (IRR=3.2, p=0.039). Adverse maternal outcomes (eclampsia, ICU admission) and fetal outcomes (low birthweight, NICU admission, stillbirth) were significantly frequent in preeclampsia (p=0.002).

**Conclusion:**

Maternal vascular lesions were significantly increased, with a 4.4-fold increase in preeclampsia and a 2.5-fold increase in gestational hypertension, compared with normotensive pregnancies. These findings highlight the central role of maternal vascular pathology in the pathogenesis of hypertensive disorders and adverse maternal and fetal outcomes. We recommend high-quality antenatal care for early detection of hypertensive disorders and placental histopathology as a valuable diagnostic tool.

## INTRODUCTION

Hypertensive disorders complicate 11.6% of pregnancies, and remain a major determinant of maternal and perinatal morbidity and mortality. These disorders include gestational hypertension, preeclampsia, chronic hypertension, and eclampsia.^1^ In Pakistan, they account for a third of maternal deaths and are associated with stillbirths, fetal intrauterine growth restriction (IUGR), preterm delivery, low birthweight (LBW), intensive care unit (ICU) admissions.^2^,^3^

The placenta reflects the health of both mother and her fetus, and its histopathological examination provides insight into the mechanisms underlying adverse pregnancy outcomes.^4^ Placental pathology is a relatively new discipline, world-wide. The Amsterdam consensus criteria (2016) classify placental pathologies into four categories: Maternal vascular malperfusion (MVM), fetal vascular malperfusion (FVM), acute chorioamnionitis (ACA), and chronic villitis of unknown etiology (VUE).^5^ MVM results from reduced maternal blood flow to placenta, causing hypoxic-ischemic villous changes, and is strongly associated with hypertensive disorders.^5^ FVM reflects impaired fetal-placental circulation caused by anomalies of the umbilical cord, fetal vessels, and uterus.^5^ ACA is inflammation of the fetal amnion and chorionic membranes typically associated with ascending infection. VUE represents maternal T cell-mediated inflammation, linked to congenital fetal viral infections.^6^

Placental histopathological examination is not routinely requested by the attending obstetricians and neonatologists in most of the hospitals, and data from Pakistan is under-represented in literature, even though the country carries a disproportionate share of South Asia’s maternal and neonatal mortality burden.^7^ To address this research gap, we aimed primarily to evaluate the types of placental lesions associated with preeclampsia and to compare these with gestational hypertension and normotensive pregnancies. As a secondary objective we compared the maternal and fetal outcomes among the three clinical groups.

## METHODOLOGY

This analytical, cross-sectional, and single-center study was conducted at the Departments of Pathology and the Gynecology & Obstetrics, Dow Hospital, Dow University of Health Sciences. Samples and data were prospectively collected using non-probability, convenience sampling, and pre-approved data collection forms, from January to December 2021.

### Ethical approval:

The study was conducted in accordance with the Declaration of Helsinki, and approved by the Institutional Review Board of Dow University of Health Sciences (IRB-1820/DUHS/Approval/2020; extended EXT2023/322 and 2025/482). Written, informed consent was obtained from all subjects or their attendants.

### Inclusion criteria:

- Pregnant women presenting to the labor room or emergency department and diagnosed with gestational hypertension, preeclampsia, eclampsia by attending obstetricians.
- Normotensive pregnant women enrolled as controls.

### Exclusion criteria:

- Multiple gestations.
- Placenta previa.
- Known maternal hematological disorders.

### Clinical definitions:

Blood pressure was measured by trained nursing staff, using automated monitors. Gestational hypertension was defined as BP>140/90mmHg, on two occasions after 20 weeks’ gestation. Severe hypertension was defined as BP>160/110mmHg.^8^ Preeclampsia was diagnosed when hypertension was accompanied with proteinuria and/or evidence of maternal organ dysfunction; while eclampsia was defined as new-onset seizures in this setting.^8^ Mothers and neonates were followed until hospital discharge.

### Maternal and neonatal outcomes:

Maternal outcomes documented included eclampsia, ICU admission, infections, readmissions and maternal death.

Neonatal outcomes included gestational age at delivery, live/stillbirth, neonatal ICU (NICU) admission, delivery-related complications, and birthweights, classified as low (<2500g), very low (<1500g), and extremely low (<1000g).^9^

### Gross and microscopic examination of the placentae:

Placentae were collected at delivery, fixed in 10% formalin, and weighed after trimming of membranes and umbilical cord. Gross examination was performed according to established guidelines.^10^ Representative sections were submitted from umbilical cord, membranes, placental disc and any grossly identified lesions. Microscopic examination was performed by two pathologists, and discrepancies were resolved by consensus. Placental lesions were classified according to the Amsterdam criteria as MVM, FVM, and inflammatory lesions.^5^ Few placentae had overlapping lesions that were classified accordingly.

### Statistical analysis:

Data were analyzed using IBM SPSS Statistics version 26. Descriptive statistics were computed for demographical and clinical data. Chi-square, Fisher exact and Monte Carlo tests were performed for categorical data. Associations between clinical groups and placental lesions was assessed using Poisson regression models for MVM, FVM, and inflammatory lesions; maternal age and gestational age were included as covariates for MVM, while diagnosis alone was included as a predictor for FVM and inflammatory lesions. The results were expressed as incidence rate ratio (IRR) with 95% confidence intervals, with p-values considered significant at <0.05.

## RESULTS

### Demographic characteristics of the study population:

A total of 76 pregnant women were included: 45 (59.2%) were booked, 25 (32.9%) presented via emergency, and 6 (7.9%) were referred from other healthcare facility. Booking status, severe hypertension and antihypertensive treatment were significantly associated with clinical group; women with preeclampsia were more likely to present as un-booked emergency cases and to have severe hypertension. Gestational age, fetal and placental weights and fetoplacental weight ratio differed significantly across groups (Table-I).

### Maternal and fetal outcomes in pregnancies:

Maternal and fetal outcomes differed substantially across clinical groups. The highest adverse outcomes were observed in preeclampsia. Maternal complications included eclampsia, ICU admissions, and maternal death, while fetal outcomes included higher rates of low birthweight, NICU admission, and stillbirths (Table-I).

**Table-I:** Demographic and Clinical Characteristics Among the Clinical Groups of Pregnancies.

Characteristics	Normotensive controls N=23 (%)	Gestational Hypertension N=24 (%)	Preeclampsia N=29 (%)	P-value
***Cases***				
Booked	20 (87.0)	19 (79.2)	6 (20.7)	29.500^M^ <0.001
Emergency (not booked)	3 (13.0)	4 (16.7)	18 (62.1)	
Referred from other health care facility	0 (0)	1 (4.2)	5 (17.2)	
***Hypertension***				
Severe	0 (0)	4 (16.7)	21 (72.4)	99.485^M^ <0.001
Not severe	0 (0)	20 (83.3)	8 (27.6)	
No hypertension	23 (100)	0 (0)	0 (0)	
***Treatment status***				
On known anti-hypertensives	0 (0)	16 (66.7)	13 (44.8)	35.856^M^ <0.001
Not on any anti-hypertensive	23 (100)	5 (20.8)	10 (34.5)	
Not known	0 (0)	3 (12.5)	6 (20.7)	
	*Mean ± SD*	*Mean ± SD*	*Mean ± SD*	
Maternal age (years)	29.5 ± 4.7	31.2 ± 4.7	28.3 ± 5.2	0.122^O^
Gestational age (weeks)	36.6 ± 1.9	36.8 ± 1.7	34.2 ± 3.4	<0.001^O^
Fetal weight (kg)	2.7 ± 0.3	2.8 ± 0.4	2 ± 0.7	<0.001^O^
Placental weight (kg)	423.6 ± 76.2	419.6 ± 91.5	342.6 ± 119.7	0.005^O^
Fetal/Placental weight	6.8 ± 1.3	7 ± 1.7	5.9 ± 0.9	0.007^O^
***Maternal Outcomes***				
No complications	22 (95.7)	21 (87.5)	15 (51.7)	24.621^M^ 0.005
Eclampsia+Maternal death	0 (0)	0 (0)	1 (3.4)	
Eclampsia + ICU	0 (0)	0 (0)	7 (24.1)	
ICU	0 (0)	1 (3.8)	4 (13.8)	
Eclampsia	0 (0)	0 (0)	1 (3.4)	
Cardiomyopathy+ ICU	0 (0)	0 (0)	1 (3.4)	
Post-surgery infection	1(4.3)	1 (4.2)	1 (3.4)	
Post-surgery RPOC	0 (0)	1 (4.2)	0 (0)	
***Fetal Outcomes***				
No complications	19 (82.6)	21 (87.5)	10 (34.5)	31.384^M^ 0.002
LBW	3 (13)	2 (8.3)	10 (34.5)	
Stillbirth	0 (0)	0 (0)	1 (3.4)	
Stillbirth + ELBW	0 (0)	0 (0)	1 (3.4)	
Stillbirth + VLBW	0 (0)	0 (0)	1 (3.4)	
Neonatal death	1 (4.3)	0 (0)	0 (0)	
NICU	0 (0)	1 (3.8)	0 (0)	
NICU + LBW	0 (0)	0 (0)	1 (3.4)	
NICU + VLBW	0 (0)	0 (0)	3 (10.2)	
NICU + ELBW	0 (0)	0 (0)	1 (3.4)	
Meconium aspiration	0 (0)	0 (0)	1 (3.4)	

M Monte Carlo Chi-square test,

O One Way ANOVA. ICU: Intensive care unit, RPOC: Retained products of conception, NICU: Neonatal Intensive care unit, LBW=Low birthweight <2500g, VLBW=Very low birthweight <1500g, ELBW=Extremely low birthweight <1000g.

### Categories of the placental lesions:

These differed significantly among the three clinical groups (Monte Carlo Chi-square 27.771, p=0.018). MVM was most frequent in preeclampsia (9/29, 31.0%). FVM was marginally more frequent in the control group (3/23, 13.0%), and acute inflammation showed similar frequencies across groups. Dual lesions: MVM and FVM, MVM and acute inflammation were more frequent in preeclampsia (Table-II).

**Table-II:** Association of Categories of Placental Lesions with the Clinical Groups of Pregnancies.

Categories of Placental Lesions	Normotensive controls N=23 (%)	Gestational Hypert-ension N=24 (%)	Preeclampsia N=29 (%)	P-value
MVM	2 (8.7)	5 (20.8)	9 (31.0)	27.771^M^ 0.018
FVM	3 (13.0)	2 (8.3)	1 (3.4)	
Acute Inflammation	4 (17.4)	4 (16.7)	3 (10.3)	
MVM+FVM	1 (4.3)	4 (16.7)	5 (17.2)	
MVM+ Acute Inflammation	0 (0)	1 (4.2)	4 (13.8)	
FVM+ Acute Inflammation	0 (0)	1 (1)	0 (0)	
MVM+ FVM+ Acute Inflammation	0 (0)	1 (4.2)	4 (13.8)	
VUE	1 (4.3)	0 (0)	0 (0)	
No placental lesions	12 (52.2)	6 (25.0)	3 (10.3)	

MVM: Maternal Vascular Malperfusion, FVM: Fetal Vascular Malperfusion,

VUE: Villitis of unknown origin,

M Monte Carlo Chi-square test.

### Histopathological Features of the Placental Lesions:

Histological features of MVM differed significantly across groups, with higher frequencies of accelerated villous maturation (AVM), distal villous hypoplasia (DVH), villous infarction, and decidual arteriopathy in preeclampsia (Table-III, Fig.1). In contrast, placental lesions of FVM and acute inflammation did not differ significantly (Table-III, Figs.2-3). However, decidual plate acute inflammation (DPAI) showed a significant increasing trend from normotensive controls to preeclampsia (p<0.001), (Table-III).

**Table-III:** Placental Lesions in Preeclampsia, Gestational Hypertension, and Normotensive Pregnancies.

Placental lesions	Normotensive controls N=23 (%)	Gestational Hypertension N=24 (%)	Preeclampsia N=29 (%)	Chi-square^¥^/Fisher exact^€^/Monte Carlo ^M^	P-value
***MVM***					
AVM	3 (13.0)	8 (33.0)	20 (69.0)	17.416^¥^	<0.001
DVH	0 (0)	1 (4.2)	7 (24.1)	8.412^€^	0.009
Villous infarction	1 (4.3)	4 (16.7)	15 (51.7)	16.532^¥^	<0.001
Decidual arteriopathy	6 (26.1)	16 (66.7)	21 (72.4)	12.658^¥^	0.002
Placental weight < 10^th^ percentile	3 (13.0)	3 (12.5)	13 (44.8)	9.837^¥^	0.007
***FVM***					
Umbilical cord compression	1 (4.3)	2 (8.3)	0 (0)	2.419^M^	0.284
Avascular villi	1 (4.3)	3 (12.5)	4 (13.8)	1.360^M^	0.507
VSVK	1 (4.3)	1 (4.2)	5 (17.2)	3.817^M^	0.164
Thrombi in fetal vessels	5 (21.7)	7 (29.7)	6 (20.7)	0.591^¥^	0.744
Fibrin in fetal vessels	3 (13)	6 (25)	8 (27.6)	1.702^¥^	0.427
Stem villous obliteration	0 (0)	1 (4.2)	3 (10.3)	2.838^M^	0.242
Vascular ectasia	1 (4.3)	2 (8.3)	2 (6.9)	0.311^M^	>0.99
***Acute Inflammation***					
Acute Chorioamnionitis	3 (13.0)	3 (12.5)	3 (10.3)	0.104^M^	0.949
Acute Villitis/ intervillositis	1 (4.3)	4 (16.7)	8 (27.6)	4.890^¥^	0.087
VUE	1 (4.3)	0 (0)	0 (0)	2.335^M^	0.307
***Additional decidual features:***					
DPAI	8 (34.8)	17 (70.9)	24 (82.8)	13.509^¥^	0.001
DPCI	3 (13.0)	3 (12.5)	4 (13.8)	4.691^¥^	0.990
DPLN	9 (39.1)	14 (58.3)	20 (69.0)	0.020^¥^	0.096

MVM: Maternal vascular malperfusion, FVM: Fetal vascular malperfusion,

VUE: Villitis of unknown etiology, AVM: Accelerated villous maturation, DVH: Distal villous hypoplasia,

VSVK: Villous stromal vascular karyorrhexis, DPAI: Decidual plate acute inflammation,

DPCI: Decidual plate chronic inflammation, DPLN: Decidual plate laminar necrosis,

¥ Chi-square test,

€ Fisher exact test,

M Monte Carlo Chi-square test.

### Poisson regression models:

Poisson regression showed a significant association between clinical groups and placental MVM lesion counts (χ²=41.75, *p*<0.001), with higher counts in preeclampsia and gestational hypertension (IRR 4.4 and 2.5, relative to the normotensive controls. Maternal age was inversely associated with MVM lesions, indicating a decline in lesion count with increasing age. No significant association was observed for FVM lesions (χ²=3.25, *p*=0.197). Acute inflammatory lesions showed a borderline association (χ²=5.32, *p*=0.070), with higher counts in preeclampsia compared to controls (Table-IV).

**Table-IV:** Association of Placental Lesions with Hypertensive Disorders of Pregnancy.

Lesion Type	Diagnosis Group	IRR (95% CI)	P-value	EM Mean ± SE
MVM	Preeclampsia	4.42 (2.45–7.97)	<0.001	2.45 ± 0.30
	Gestational hypertension	2.51 (1.32–4.80)	0.005	1.39 ± 0.25
	Normotensive controls	1.0	–	0.56 ± 0.15
	Maternal Age (per year)	0.96 (0.93–1.00)	0.039	–
FVM	Preeclampsia	1.72 (0.87–3.41)	0.121	0.90 ± 0.18
	Gestational hypertension	1.76 (0.87–3.55)	0.116	0.92 ± 0.20
	Normotensive controls	1.0	–	0.52 ± 0.15
Acute Inflammatory Lesions	Preeclampsia	3.17 (1.06–9.49)	0.039	0.90 ± 0.18
	Gestational hypertension	1.92 (0.58–6.37)	0.288	0.92 ± 0.20
	Normotensive controls	1.0	–	0.52 ± 0.15

MVM: Maternal Vascular Malperfusion, FVM: Fetal Vascular Malperfusion, IRR: Incidence Rate Ratio,

EM: Estimated Marginal, SE: Standard error.

## DISCUSSION

A total of 76 placental specimens were analyzed, including cases of preeclampsia (n=29), gestational hypertension (n=24), and normotensive controls (n=23). Histopathological features of MVM differed significantly among the three clinical groups, and Poisson regression revealed a 4.4-fold increase in MVM lesion count in preeclampsia and a 2.5-fold increase in gestational hypertension, compared with controls. In contrast, no significant differences were observed for FVM lesions. Acute inflammatory lesions showed a marginal association, with preeclampsia presenting a 3.2-fold higher lesion count. Preeclamptic pregnancies also showed the highest rates of maternal and fetal complications, including eclampsia, ICU/NICU admissions, low birthweight, three stillbirths and one maternal death.

Incomplete spiral artery remodeling and chronic placental oxidative stress, the hallmark pathophysiology of MVM, manifest histopathological as decidual arteriopathy, accelerated villous maturation, distal villous hypoplasia and infarction.^11^ All of these MVM findings were significantly more frequent in preeclamptic placentae in our study. These findings are consistent with previous studies reporting a high incidence of MVM in preeclampsia.^12^,^13^ Defective maternal immune response, shallow trophoblastic invasion and Vitamin-D deficiency have been proposed as contributing factors to defective placentation and MVM.^14^,^15^ The trophoblast releases proinflammatory, prothrombotic, and vasoconstrictive cytokines into the maternal circulation, leading to the maternal manifestations of preeclampsia: hypertension, low platelets, and end-organ damage.^16^ MVM has been identified as an independent risk factor for adverse fetal outcomes including growth restriction, LBW, prolonged hospital admission and intracerebral hemorrhage.^17^ Significant reductions in fetal and placental weights observed in preeclampsia are consistent with the disease pathophysiology and have been documented in the literature.^18^

FVM results from restricted blood supply to the fetus through the placenta, and manifests as fibrin thrombi in fetal vessels, avascular villi, villous karyorrhexis, villous edema and delayed villous maturation.^5^ FVM has been linked to maternal disseminated intravascular coagulation, thrombophilia and antiphospholipid antibodies, and is associated with an increased risk of fetal brain injury and neurodevelopmental impairment in infants.^19^ Several of our cases of preeclampsia showed placental lesions of FVM along with MVM, which suggests that fetal vessel thrombosis is possibly a late complication in preeclampsia, after the activation of the coagulation cascade.^20^ Comparable findings have been reported in neonates diagnosed with growth restriction, where preeclampsia was identified as an independent risk factor for combined lesions of MVM and FVM.^21^ Similarly, another study reported a high incidence of MVM and FVM lesions in placentae analyzed from stillbirths and preterm neonatal deaths.^22^ Studies on acute chorioamnionitis in preeclampsia have been inconsistent, with some studies showing a significant association, while others reporting none.^13^,^23^

We also noted that inflammatory lesions were significantly increased in preeclampsia, suggesting that maternal and fetal malperfusion and inflammation are interconnected, rather than mutually exclusive, and possibly act synergistically to drive the adverse maternal and fetal outcomes.

In our study, women with preeclampsia were more likely to present as emergency cases with severe hypertension, underscoring the importance of antenatal care. Current recommendations advise at least eight antenatal visits with a healthcare provider during pregnancy.^24^ High-quality antenatal care can identify high-risk cases of hypertension, enable early prevention, diagnosis and treatment, and thereby prevent complications of maternal organ damage, eclampsia, stillbirth, preterm birth and neonatal complications.^25^ In Pakistan, however, fewer than half of pregnant women receive adequate quality and quantity of antenatal health care.^25^

We therefore recommend regular antenatal follow-up for early detection and surveillance of preeclampsia. We suggest that obstetricians and neonatologists request histopathological examination, and microbiological culture of placentae, in cases of preeclampsia, preterm delivery, stillbirth and neonatal death, and in fetuses suspected of thrombotic disorders and infections, to assess the extent of placental lesions and possible cause of fetal demise.

### Strengths of the study:

The application of the standardized Amsterdam classification, comprehensive statistical analysis across clinical groups, and correlation of histopathological findings with adverse maternal and fetal outcomes.

### Limitations

The study had a single-center, cross-sectional design, a small sample size and potential selection bias. Microscopic examination was unblinded, which may have introduced an observer bias. Additionally, microbiological culture of the placental tissue could not be performed to confirm suspected infections.

## CONCLUSION

Maternal vascular lesions were significantly increased, with a 4.4-fold increase in preeclampsia and a 2.5-fold increase in gestational hypertension, compared with normotensive pregnancies. These findings highlight the central role of maternal vascular pathology in the pathogenesis of hypertensive disorders and adverse maternal and fetal outcomes. We recommend high-quality antenatal care for early detection of hypertensive disorders and placental histopathology as a valuable diagnostic tool.

### Recommendations

Further research should focus on large-scale, multi-center cohort studies examining MVM, FVM and inflammatory placental lesions in hypertensive disorders and their predictive value and long-term effects on maternal and offspring health.

## Availability of data and materials:

Data is available at: http://doi.org/10.6084/m9.figshare.30610700

## References

[1] Lei J, Zhao M, Li L, Ji B, Xu T, Sun M (2022). Research progress of placental vascular pathophysiological changes in pregnancy-induced hypertension and gestational diabetes mellitus. Front Physiol.

[2] National Institute of Population Studies (NIPS) [Pakistan] and ICF 2020. 2019 Pakistan Maternal Mortality Survey Summary Report. Islamabad, Pakistan, and Rockville, Maryland, USA: NIPS and ICF.

[3] McClure EM, Saleem S, Goudar SS, Tikmani SS, Dhaded SM, Hwang K (2022). The causes of stillbirths in south Asia: results from a prospective study in India and Pakistan (PURPOSe). Lancet Glob Health.

[4] Baergen RN (2007). The placenta as witness. Clin Perinatol.

[5] Redline RW, Ravishankar S, Bagby CM, Saab ST, Zarei S (2021). Four major patterns of placental injury: a stepwise guide for understanding and implementing the 2016 Amsterdam consensus. Mod Pathol.

[6] Jain VG, Willis KA, Jobe A, Ambalavanan N (2022). Chorioamnionitis and neonatal outcomes. Pediatr Res.

[7] Liu B, Huang X, Hao Z, Wang J, Fan Y, Shao Q Global burden of maternal hypertensive disorder from 1990 to 2021. medRxiv.

[8] Garovic VD, Dechend R, Easterling T, Karumanchi SA, McMurtry Baird S, Magee LA (2022). Hypertension in pregnancy: diagnosis, blood pressure goals, and pharmacotherapy: a scientific statement from the American Heart Association. Hypertension.

[9] Okwaraji YB, Krasevec J, Bradley E, Conkle J, Stevens GA, Gatica-Domínguez G (2024). National, regional, and global estimates of low birthweight in 2020, with trends from 2000: a systematic analysis. Lancet.

[10] Roberts DJ, Baergen RN, Boyd TK, Carreon CK, Duncan VE, Ernst LM (2023). Criteria for placental examination for obstetrical and neonatal providers. Am J Obstet Gynecol.

[11] Baergen RN (2011). Chapter: Early placental development. In Manual of pathology of the human placenta. Second ed., Springer Science & Business Media: New York, USA,.

[12] Zur RL, McLaughlin K, Aalto L, Jiang Y, Huszti E, Parks WT (2024). Phenotypes of maternal vascular malperfusion placental pathology and adverse pregnancy outcomes: A retrospective cohort study. BJOG.

[13] Yogeshkumar S, Dhananjay S, Gowdar S, Gowdar G, Kulkarni V, Byranahalli S (2023). Morphological study of the placenta in deliveries with pre-eclampsia: Results from a prospective, observational study in India and Pakistan (PURPOSe). BJOG.

[14] Staff AC, Fjeldstad HE, Fosheim IK, Moe K, Turowski G, Johnsen GM (2022). Failure of physiological transformation and spiral artery atherosis: their roles in preeclampsia. Am J Obstet Gynecol.

[15] Ali S, Huma Z, Yasmin H, Hussain S (2024). Vitamin-D deficiency as a potential indicator of defective placentation in preeclampsia. Pak J Med Sci.

[16] Brosens I, Puttemans P, Benagiano G (2019). Placental bed research: I The placental bed: from spiral arteries remodeling to the great obstetrical syndromes. Am J Obstet Gynecol.

[17] Jiang H, Shi D, Cai L, Zhang Y, Sun Q, Jiang H (2025). Characteristics of maternal-placental vascular malperfusion and its correlation with neonatal adverse outcomes. J Repro Immunol.

[18] Freedman AA, Suresh S, Ernst LM (2023). Patterns of placental pathology associated with preeclampsia. Placenta.

[19] Spinillo A, Dominoni M, Dal Mas F, Cesari S, Fiandrino G, Gardella B (2023). Placental fetal vascular malperfusion, neonatal neurologic morbidity, and infant neurodevelopmental outcomes: a systematic review and meta-analysis. Am J Obstet Gynecol.

[20] Ravikumar G, Mascarenhas D, Suman Rao PN, Crasta J (2022). Fetal vascular malperfusion (FVM): diagnostic implications and clinical associations. J Matern Fetal Neonatal Med.

[21] Shmueli A, Mor L, Blickstein O, Sela R, Weiner E, Gonen N (2023). Placental pathology in pregnancies with late fetal growth restriction and abnormal cerebroplacental ratio. Placenta.

[22] Kulkarni VG, Sunilkumar KB, Nagaraj TS, Uddin Z, Ahmed I, Hwang K (2021). Maternal and fetal vascular lesions of malperfusion in the placentas associated with fetal and neonatal death: results of a prospective observational study. Am J Obstet Gynecol.

[23] Stanek J, Funk D (2025). Clinicopathologic correlation and interdependence of basic patterns of placental injury. Virchows Arch.

[24] World Health Organization (2022). WHO antenatal care recommendations for a positive pregnancy experience. Maternal and fetal assessment update: Imaging ultrasound before 24 weeks of pregnancy. World Health Organization;.

[25] Neupane S, Scott S, Piwoz E, Kim SS, Menon P, Nguyen PH (2023). More is not enough: High quantity and high quality antenatal care are both needed to prevent low birthweight in South Asia. PLOS Glob Public Health.

